# GenoDense-Net: unraveling the genomic puzzle of the global pathogen

**DOI:** 10.1186/s40794-025-00267-y

**Published:** 2025-09-02

**Authors:** Shivendra Dubey, Sakshi Dubey, Kapil Raghuwanshi, Pranshu Pranjal, Sudheer Kumar

**Affiliations:** 1 Department of Artificial Intelligence & Machine Learning, Manipal University Jaipur, Jaipur, India; 2 Department of Electronics and Communications, RKDF University, Bhopal, MP India; 3 IcfaiTech (Faculty of Science and Technology), The ICFAI University, Jaipur, Rajasthan, Jaipur, India; 4 Department of Artificial Intelligance & Machine Learning, Manipal University Jaipur, Jaipur, Rajasthan India; 5 Department of Computer Science and Engineering, University of Engineering and Management, Jaipur, Rajasthan India

**Keywords:** DenseNet-16, NearKbest, Adam optimizer, VGG-16, Genome sequence

## Abstract

The respiratory system of humans is impacted by infectious and deadly illnesses like COVID-19. Early identification and diagnosis of this type of illness is essential to stop the infection from spreading further. In the present research, we presented a technique for determining the condition using COVID-19's current genome sequences employing the DenseNet-16 framework. We operated a network of already trained neurons before using a transfer learning method to prepare it according to our dataset. Additionally, we preprocessed the collected information using the NearKbest interpolation approach; then, we utilized Adam Optimizer to optimize our findings. Compared with special deep learning models like ResNet-50, VGG-19, AlexNet, and VGG-16, our approach produced an accuracy of 99.18%. The model was deployed on a platform with GPU support, which greatly decreased training time. Dataset size and the requirement for further validation are two of the study's limitations, despite the encouraging results. The current research showed how a deep learning approach may be useful to categorize the genome sequence of infectious disease like COVID-19 using the suggested GenoDense-Net architecture. The next step in this research project is conducting investigations in the clinic.

## Introduction

A crucial task in comprehending the complex genomic makeup of infectious disease like COVID-19 is unraveling the worldwide pathogen's genomic puzzle. An extraordinary global effort by researchers and scientists is underway to interpret the viruses'genetic coding, reconstruct their genomic design, and trace their history of evolution. This unrelenting search intends to reveal critical insights about the transmission dynamics, mutation patterns, and the virus's origin through thorough genome analysis and sequencing. By solving the genomic riddle, we can learn crucial details about the potential variants and behavior of the virus, which will help us design specialized diagnostic equipment, therapies, and preventative measures. In addition to being a scientific accomplishment, this quest holds the potential to inform public health policies and direct our group's reaction to the global epidemic. The corona virus is an infectious disease, also called COVID-19, was discovered for the first time in the final few days of 2019. That expressed how critically alarming the circumstance globally referred to an international epidemic. This illness impacts the respiratory system of humans [1, 2].

Given the numerous sufferers, these available kits for diagnosis of infectious disease were insufficient because they failed to recognize every instance. Therefore, the researchers found other ways of diagnosing the viral infection using the patient's CT scan (Computerized Tomography scan) images from their lungs. Most people cannot afford the astronomical cost of CT scans. Therefore, we use genomic sequence analysis of infectious disease as the foundation of our technique [3].

### Role of genome sequence

By revealing vital information about the biological makeup of the microorganisms accountable for the diseases, the sequencing of genomes serves a critical role in diagnosing infectious diseases. The following represent a few crucial functions of the sequencing of genomes in the detection of infectious diseases:

#### Pathogen identification

DNA sequencing enables researchers to ascertain a pathogen's unique genetic signature, allowing for precise identification. The bacterium causing the sickness is identified by matching a pathogenic microbe's genetic makeup to a repository containing known sequences [4].

#### Transmission dynamics understanding

Genome sequencing aids in tracing the paths of infectious disease transmission, which is essential for understanding transmission dynamics. Scientists can track the clusters of related cases and information on the disease by comparing genomes of viruses isolated from various people or geographic areas. For conducting specific measures and managing epidemics, this information is essential [5].

#### Drug resistance detection

Drug resistance detection is made easier with the help of genome sequencing for infections. Researchers can pinpoint genetic variations linked to antimicrobial medication resistance by analyzing the genomic sequence. This information helps decide on the best course of action for treating patients and creating plans for preventing the spread of strains that are resistant to medicines [6].

#### Development of vaccine

Genome sequencing is essential for the creation of vaccinations against diseases that are infectious. A pathogen's genetic structure is examined to discover important antigens and create vaccines specifically designed to elicit a response from the immune system. This method has proven to be especially helpful in quickly creating vaccinations for contagious diseases like COVID-19 [2].

#### Epidemiological research

The sequencing of genomes aids epidemiological study by supplying necessary information on the molecular evolution and modes of transmission of infections. To develop control and efficient preventative measures, researchers can learn more about the source, propagation, and development of infectious illnesses by examining gene variations in the pathogen population [7].

### Genome classification techniques based on AI

Transformer-based frameworks like DeepSequence, GenomeBERT, and BERT for DNA have been investigated in recent developments in AI-driven genomic classification. These models perform better in predicting mutation effects and classifying regulatory sequences by using attention processes to capture contextual linkages and long-range interactions within genomic sequences. Future research should investigate the incorporation of this type of transformer-based genome frameworks for more accurate and multimodal COVID-19 diagnosis, even if our present study has focused on CNN-based feature selection and extraction with chest scans.

GenomeBERT—Better results on tasks involving the annotation of genomes and the prediction of mutation effects are made possible by a customized version of BERT that was previously trained on individual DNA sequences.

BERT for DNA—The BERT architecture is modified to sequences of DNA using a contextual embedding approach to capture functional and regulatory patterns.

DeepSequence—A computational model that infers variation consequences of substantial biological significance by using unsupervised learning from numerous sequence matches.

### Objectives of the research

Every research has some objectives to develop innovative ideas. This study work on the proposed model with having some objectives these are:

• To understands the vital necessity of promptly identifying in preventing the transmission of infectious virus, a severe and extremely transmissible respiratory disorder.
• To creates an approach for identifying infectious disease like COVID-19 in individuals'X-ray images of the chest. We can see this approach with the DenseNet-16 framework to be the foundation.
• To use a neural network those was trained and hone it on the particular dataset to utilize techniques for transfer learning. To use the Nearest-Neighbor approximation approach to process the chest X-ray pictures in order to make them more analytically suitable.
• To use the Adam Optimizer for improve the framework's diagnostic accuracy by optimizing its parameters.
• To compare the proposed framework with various deep learning frameworks like ResNet-50, AlexNet, VGG-19, and VGG-16 the level of precision gained is better. That demonstrate the suggested DenseNet-16 based technique works better than various competing models.

## Literature review

By thousands of researchers worldwide have been collected information and creating strategies to reveal the epidemiological, pathogenetic, and medical characteristics of SARS-CoV-2. Artificial intelligence has been used to expedite treatment development, forecast the spread of the virus, and diagnose patients. A preliminary diagnostic model and RT-PCR (Reverse Transcription-Real Time Polymerase Chain Reaction) and employing deep learning approaches with lung CT scans (computed tomography scans) has been compare in an investigation aimed at identifying COVID-19 infection [8]. From lung computed tomography (CT scans) sets, a 3-D deep learning algorithm was created, employing 618 CT observations. Implemented a location-attention classification approach, the machine trained to distinguish between pictures and classify them into non-infectious, viral pneumonia, COVID-19, and Influenza-A [9]. Considering a median success rate of 86.7 per cent based on the CT scans utilized, the deep learning models successfully identified COVID-19 patients. It has been employed of Transcription-Real Time Polymerase Chain Reaction (RT-PCR) tests as the norm for infectious disease recognition [3]; causes significant delays in identifying suspicious people, creating several unique obstacles to the prevention of the disease. The COVID-19 detection investigation included an initial collection of 540 samples from patients (COVID-negative and COVID-positive) [10]. Of the total, 229 and 313 patients using the samples had negative and positive results.

For identifying COVID-19 using CT scans, a three-dimensional DeCoVNet (Deep Convolutional Neural Network) has been developed. Each step comprises the network stem (the DeCoVNet), two 3-dimensional residual blocks, including as a ProClf (Progressive Classifier). The Progressive Classifier is where the forecast findings, come on or after. After extracting the data from the CT images, it directly generates the probability of becoming COVID-negative and COVID-positive. The analysis confirmed that the method was a shallow learning system with inadequate supervision. It did, however, achieve a high COVID-19 detection effectiveness. In numerous computational biology, computer vision, bioinformatics, and investigations, deep learning frameworks has been used to predict and categorize RNA and DNA-binding specificity, among other tasks [11]. To develop a signal detection system that recapitulates referred to motifs, a DeepBind; for example, used just one level of convolution to operate in a CNN (Convolutional Neural Network) design, while looked into additional variables in structures [12], which included the variety of layers and activities like the pooling process. Other research has employed more intricate designs incorporating all the RNN (Recurrent Neural Network) and CNN layer models, like DanQ [13] and iDeepS [14]. In an additional investigation, a bi-GRUs (bidirectional Gated Recurrent Units) layer was used as part of the KERGU technique [15] (an utterly RNN-based design. To give the network a state within it that enables it to recognize long-distance dependence, this pair an embedded model of the sequence of inputs using the KERGU algorithm [8].

In recent years, hybrid AI approaches that combine deep learning with evolutionary and metaheuristic optimization techniques have been studied. To illustrate their possible relevance to genomic categorization and mutation detection, hybrid models have been used to improve results in tasks for feature selection [16–18] and imaging for medicine [19]. Furthermore, for better disease diagnostics using genomic sequences, deep neural networks can be combined with computational evolutionary principles [20] to provide strong optimization techniques (Table 1).

**Table 1:** An overview of hybrid and current AI-based models for healthcare imaging and genome issues

Model	Pertinence to the Classification of Genomes	Major Contribution	Application Area
**Differential automatic encoder **[21]	**Very important for diagnosing diseases based on mutations**	**Sequence distributions for learning unsupervised models**	**Prediction of mutation effects**
**Frameworks for cybersecurity powered by AI **[18]	**Adaptable to safe pipelines for genomic data**	**established foundations for intelligent edge AI to make decisions quickly and securely**	**Security of medical data and edge computing**
**Transformer-based model **[22]	**The latest advancements in contextual genomic feature learning**	**BERT model pre-trained for DNA data**	**Classification of genomic sequences**
**Evolutionary metaheuristics **[19]	**Beneficial for deep genomic model parameter tweaking**	**A thorough analysis of metaheuristic algorithms**	**Genetics and engineering optimization**
**Paradigms for evolutionary computing **[23]	**Provides a foundation for using PSO/GA in analysis of sequences**	**Examining evolutionary tactics in practical issues**	**General tasks for AI optimization**
**Hybrid metaheuristics combined with feature selection **[24]	**Useful for lowering the dimensionality of genetic data**	**Enhanced categorization by the selection of feature subsets**	**Genomic analysis and machine learning**
**Optimization of hybrid deep learning **[20]	**Gives instructions on how to use hybrid optimization for genome-based DL projects**	**Suggested a new hybrid model that enhances DL performance**	**Imaging techniques for medicine (such as X-rays and CT)**
**Genomic variation analysis combined with deep learning **[25]	**Extremely pertinent to detecting and classification of viral mutations based on genomes**	**Model for detecting COVID-19 variants that is specific to mutations**	**Classification of variants of SARS-CoV-2**
**Transformers of vision and transfer learning **[26]	**Demonstrates the promise of transformer-based techniques for imaging problems related to DNA and genomics**	**Adaptation of Vision Transformer for Medical Diagnostics**	**X-rays for the identification of disease**
**Attention-focused LSTM-CNN **[27]	**High applicability for contextually aware modelling of sequential genomic data**	**Improved sequence classification performance with a hybrid model**	**Classification of DNA sequences**

### Significance of review

While this study shows the promise of artificial intelligence in infectious disease diagnosis like COVID-19, there is still a need for more thorough medical verification that is essential for putting these algorithms to use in actual clinical settings.

#### External verification

The study makes absence of outside verification that involves evaluating the hypothesis using information gathered from different medical facilities or geographic areas. To evaluate the frameworks the ability to generalize this is crucial.

#### Small-scale dataset

The utilization of a comparatively modest dataset represents a possible study need. The precision found in this dataset is encouraging, but the validation process frequently calls for accessibility to a larger, diversified dataset that is typical for an entire population.

#### Therapeutic significance

Although the research's aim is accurate diagnosis, validation in clinical settings should consider the effects of the test's findings on actual patients. This entails assessing the simulation's specificity, sensitivity, negative and positive prognostic standards, as well as its capacity to support medical experts to generate wise judgments.

#### Ethics

The study might not have addressed issues like confidentiality of patients, consent that is informed, and transparency in decision-making that are connected to utilizing artificial intelligence (AI) models in healthcare settings.

#### Reliability and robustness

A high degree of precision is important, but medical testing should also take into account the system's dependability and durability under various settings, changes in picture quality, and outside influences.

## Densenet-16 architecture

"DenseNet-16"refers to a specific design called DenseNet with 16 layers. Densely Connected Convolutional Networks (DenseNet), referred to known as deep neural network architecture [28].

Each layer in DenseNet connects to each subsequent level according to a feed-forward fashion, creating a network of dense relationships among groups. This connection architecture offers more immediate data flow and improved gradient propagation to address the disappearing gradient issue and encourage feature reuse.

**Fig. 1:**
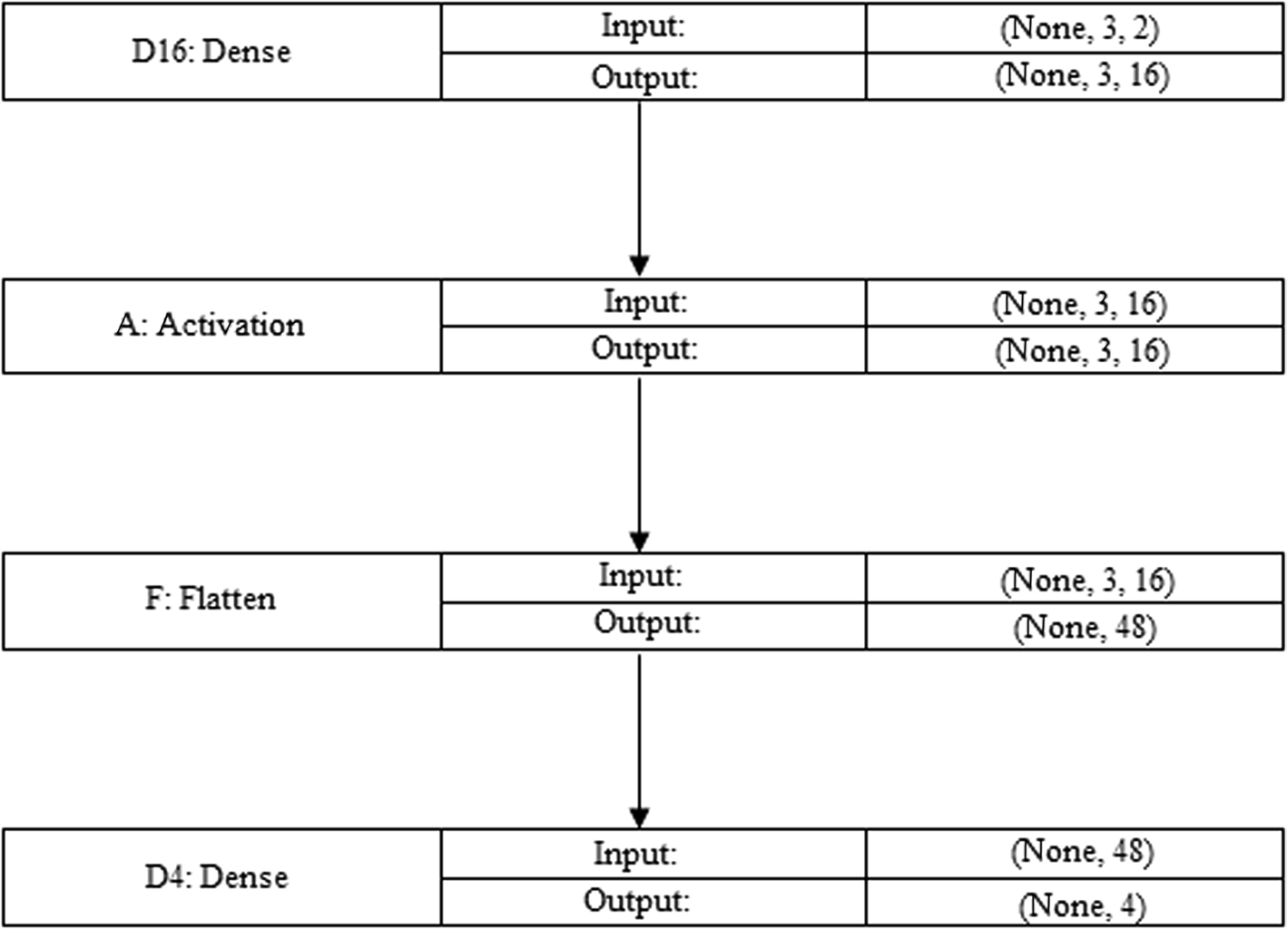
DenseNet16

The number after"DenseNet"indicates the network's layers total counts that includes fully connected layers, pooling, and convolutional layer. The instance of DenseNet-16 shows there are sixteen distinct layers in a network [29]. A top classification layer, transition layers, and dense blocks are standard components of the DenseNet design. Multiple layers make up dense blocks, and each layer inside a block has linked with each subsequent layer through that block. Reduced channel counts within dense blocks are achieved by using transition layers to regulate the dimensions of space. A fully linked layer comprising the resultant prediction common comes after a layer that pools worldwide averages as the final classification layer. Various tasks related to computer vision include picture classification, object identification, and semantic segmentation. DenseNet has demonstrated good performance. Depending on the precise needs of the work, the level of detail of the dataset, and the available computer resources, DenseNet-16 (see Fig. 1) or any additional format may be used.

## Proposed methodology

**Fig. 2:**
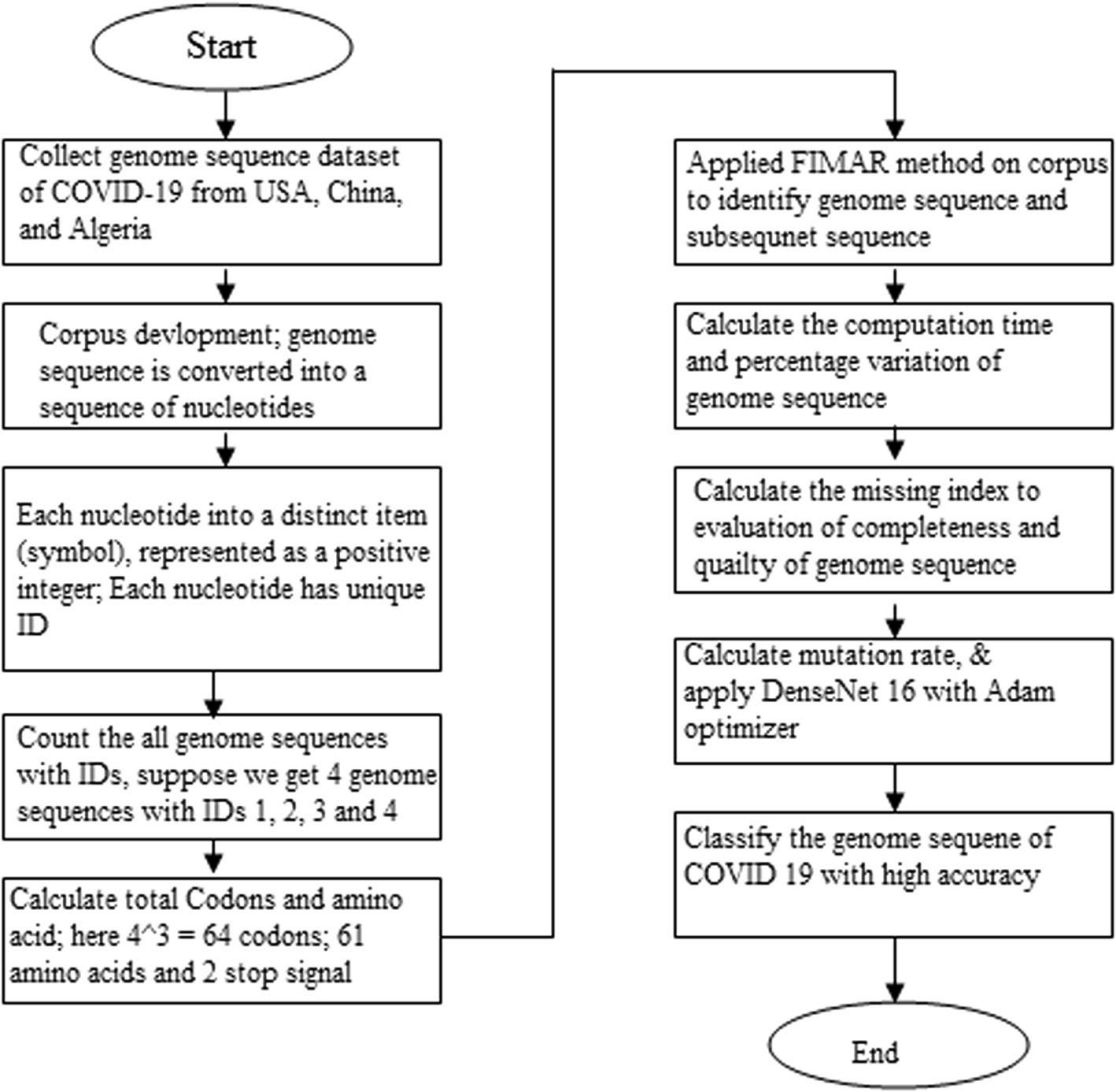
GenoDense – Net model

In this work, we have used two genome sequencing data sets are employed, including from China and the USA. We generate a frame to read every line from the provided dataset, including the line number and sequence. Beginning has been from the algorithm's search for a matched sequence. The technique for uncovering genetic code patterns is used to identify matching sequences. To calculate the overall time complexity of the GenoDese-Net model (see Fig. 2); we have identify the beginning and finishing time of the running algorithm. However, we are also use an algorithm to identify the missing sequencing and the fraction of altered genome sequences in the provided sequences.

Dense(16) indicates that there will be 16 neurons in the layer. Every single neuron within an extremely dense layer is completely associated with every neuron in the layer before it; meaning that every neuron in the layer getting information from every neuron which is in the layer before. In this model, we are using dense layers with the sigmoid activation function. This layer may describe irregularities within the data since the sigmoid function is a function that converts each neuron's output to a number that ranges from zero to one. Hyperparameter tweaking and experimentation are frequently required to determine a dense layer's ideal number of neurons. The trade-off between generalize performance and model capacity relies on the particular issue, the complexity and amount of the dataset, and other factors. Determining the optimum amount of neurons to get the required performance of the model may be done using methods like model validation and cross-validation.

The complexity and capacity of a neural network are influenced by the total amount of neurons within its dense (completely interconnected) layer. The total number of neurons represents a crucial hyperparameter that could impact the network's functioning and capacity for learning. The mathematical expense of inference and training rises as the density of a layer of neurons also increases. Resource contexts constrain the model deployment, and the need for additional neurons may impact training duration since they demand more processing capacity. The num_words of number of neurons will be present in the output layer. The num_words variable represents the classification problem's variety of categories or classes. Each neuron in the output layer denotes a certain class, as well as the chance or probability that what is input falls under that group of neurons. The softmax function as used as activation for the output layer. The outputs of the neurons in the output layer are normalised by the softmax function to ensure that they add to 1. Generating probabilities for each class in multi-class classification tasks is standard practice.

1 $$\uptheta \hspace{0.17em}=\hspace{0.17em}\uptheta -\text{learning}\_\text{rate }*\text{ m }/ (\text{sqrt}(\text{v})\hspace{0.17em}+\hspace{0.17em}\text{epsilon})$$

Here, θ stands for the neural network's parameters (biases and weights), the parameter updates'magnitude is controlled by learning_rate, often known as learning rate or step size, the gradients'first moment estimate (mean), denoted by the letter m, and the gradients'uncentered variance is represented by v, the second moment estimate. To maintain numerical stability and prevent division by zero, a minor constant called epsilon was included. We used adom optimizer method with weight decay (L2 regularisation) and a learning rate of 0.001 value of 1e-5 is used as the loss function. To minimize the loss function, the optimizer must change the weights of the model during training. That is the thing whose loss function is categorical cross-entropy. When the objectives are one-hot encoded in multi-class classification settings, categorical cross-entropy is typically used.

To set up TensorFlow and Keras, optimization on the tool must be used during training for the compilation stage of a model of neural networks. Using tf.kera optimizers, the optimizer entity it was previously generated has been referred to via opt variable Adam.

2 $$\uptheta \hspace{0.17em}=\hspace{0.17em}\uptheta -\text{learning}\_\text{rate }* \nabla \text{f}(\uptheta )$$

The SGD (stochastic gradient descent) update rule for a neural network's parameters (θ), learning rate (learning_rate), and (∇f(θ)) gradient of its loss function (f) with regard to those variables. In order to minimise the loss function, the optimizer must modify the model weights depending upon the calculated gradients. The measurement parameter is set to"accuracy,"which gauges how many outputs of all samples were properly predicted. It indicates how accurately the model is functioning in terms of categorization. The package would determine the implementation and function of the class or DNA module. It is challenging to describe the function of the DNA module or class precisely without further details about the particular package and its contents. However, the class or module may be connected to the analysis of DNA sequences or modification just on the term"DNA,"based on the name alone. It might include features for managing DNA sequences, running procedures on performing DNA or DNA data, and analysis-specific algorithms. It would be essential to consult the package's documentation or source code or request more details from its creators or maintainers to better comprehend the DNA class or module and its functionality.

**Figure:**
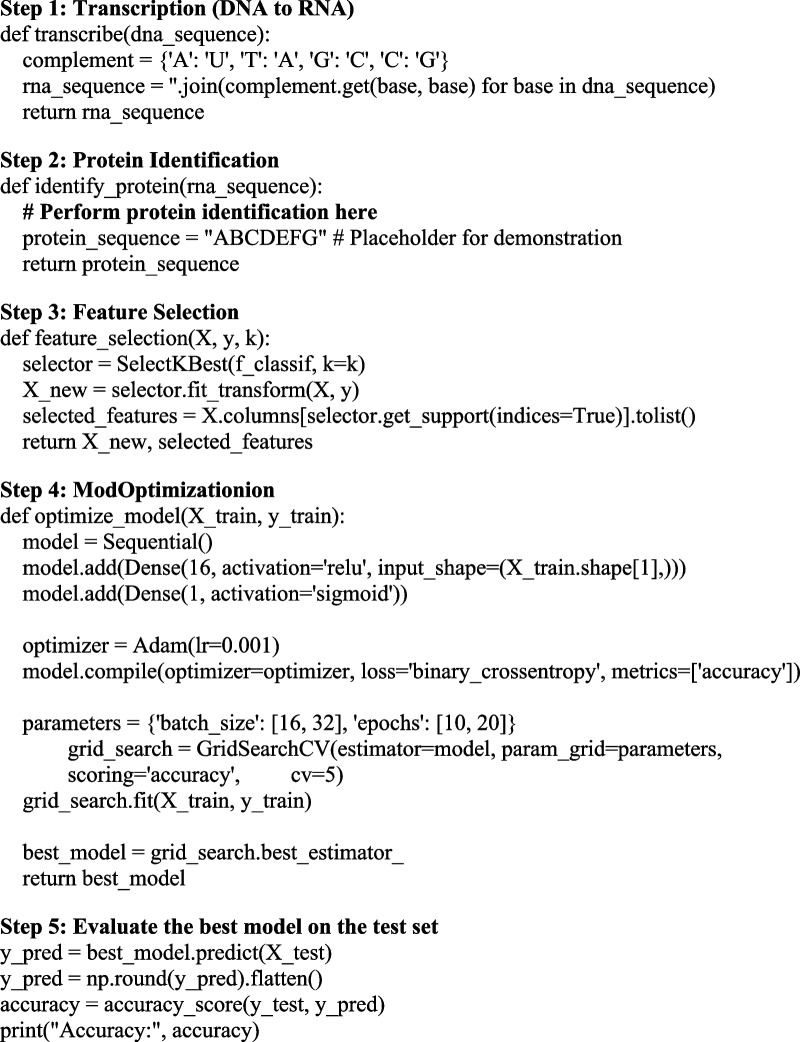
Algorithm 1 Feature selection, protein identification, transcription and model improvement

Feature selection selects the most relevant features from a wider set to enhance the model's accuracy and comprehension in Algorithm 2. Protein identification, essential for comprehending systems of biology and researching diseases, involves identifying the existence or authenticity of protein in a specimen from the body. Protein synthesis and gene expression depend heavily on transcription, which creates RNA from DNA. Model enhancement seeks to enhance the accuracy of forecasting algorithms by modifying their design, adjusting hyperparameters, or adding new data, ultimately producing more precise and insightful results forecasts.

A well-liked library for Python's bioinformatics and computational biology is the Bio package, occasionally referred to as Biopython. These offer several features for interacting with biological data, such as protein sequences, RNA, and DNA. The Biopython SeqUtils package includes utility and class methods for analyzing sequences. This instance imports the ProtParam module with SeqUtils. The ProtParam module offers methods and characteristics for computing different protein sequence features. It has characteristics to determine the molecular weight, physicochemical protein characteristics, isoelectric point, and content of amino acids. For the characterization and analysis of protein sequences, these characteristics are helpful. Functionalities about feature selection methods are included in the scikit-learn feature selection module, intending to optimize, promote interpretability, lower overfitting, and model performance. A feature selection technique centred on univariate tests of statistical significance is implemented by the SelectKBest module in the sci-kit-learn library. According to a predetermined scoring mechanism, the most outstanding K features containing the greatest score are chosen.

3 $$\text{F}=\frac{\frac{\sum_{l-1}^{n}\left(\frac{{TG}_{l}^{2}}{{N}_{l}}\right)- \left(\frac{{T}^{2}}{N}\right)}{n-1}}{\frac{\sum_{l-1}^{n}\sum_{m-1}^{{N}_{l}}{(O}_{lm}^{2})- \sum_{l-1}^{n}\left(\frac{{TG}_{l}^{2}}{{N}_{l}}\right)}{N-n}}$$

4 $$\text{MSE}=\frac{\sum_{l-1}^{n}\sum_{m-1}^{{N}_{l}}{(O}_{lm}^{2})- \sum_{l-1}^{n}\left(\frac{{TG}_{l}^{2}}{{N}_{l}}\right)}{N-n}$$

5 $$\text{MSE}=\frac{\sum_{l-1}^{n}\left(\frac{{TG}_{l}^{2}}{{N}_{l}}\right)- \left(\frac{{T}^{2}}{N}\right)}{n-1}$$

where F represents the proportion of variance across the whole test, the mean square error represent by MSE, which is calculated due to error (within residual mean square, groups), the mean square owing to groups or treatments (among all groups) represent by MST, Olm represents an observation, T is the sum of the totals of every one of observations, TGi is the group's total, N is the total amount of observation, and Nl is the sample size in group l.

The F-value of the ANOVA is among the intended classification task of target variable and each feature. It provides the *p*-value and F-value for assesses and each feature how linearly dependent each characteristic is on the target variable.

The LSTM (Long Short-Term Memory) is a sort of the RNN layer that belongs to the recurrent neural network family. When the input information sequence is crucial, LSTM layers are frequently used to handle sequential data, especially text or time series. Long-term dependencies are captured by LSTM layers, which also deal with the classic RNN's vanishing gradient issue. In a neural network, dense denotes a completely linked layer. Each of the neurons in the present coat is associated with every other neuron in the layer that follows below via dense layers. They are employed in learning non-linear mapping amongst data from both input and output. We used dropout, a regularisation method, to stop overfitting in neural networks. The network must acquire more resilient and generic representations because dropout layers arbitrarily remove an input unit's percentage through training. This help to reduce the interdependencies between neurons.

**Figure:**
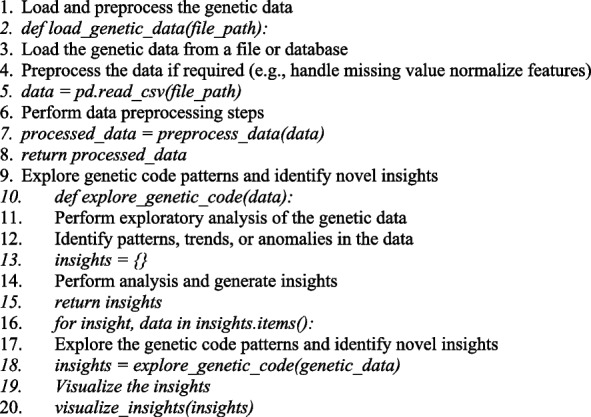
Algorithm 2 Unknown territory and uncovering new insights: unveiling genetic code patterns

The second algorithm shows how to derive new insights and investigate genetic data. It is a streamlined framework, which can develop and change according to the type of biological information we're using and our unique needs. By applying nearest-neighbor interpolation, a straightforward but efficient method that chooses the pixel count that represents the closest neighbor while scaling, the chest X-ray pictures have been expanded to 224 × 224 × 3. Because it can preserve the edge properties of healthcare pictures and has a cheap processing cost—two factors that are essential for accurate classification—this method was chosen. This interpolation technique achieves the required multidimensional integrity for DenseNet-16 without producing interpolated objects of value, which is important because our work concentrates on graphical COVID-19 detection.

### Dense blocks: feature transformation

Through feature map concatenation, each layer in DenseNet takes input from all preceding layers, not just the one immediately before it. If y represents the l^th^ layer's output, then one transformation is as follows:

6 $${\text{y}}_{\text{m}}\hspace{0.17em}=\hspace{0.17em}{\text{H}}_{\text{m}}([{\text{y}}_{0}, {\text{y}}_{1}, {\text{y}}_{2}, \dots .{\text{y}}_{\text{m}-1}])$$

H_m_(.) = Composite function which includes; ReLU activation, BN (Batch Normalization), Convolution operation.

7 $${\text{H}}_{\text{m}}\left(\text{y}\right)\hspace{0.17em}=\hspace{0.17em}{\text{V}}_{\text{m}}*(\text{ReLU}(\text{BN})(\text{y})$$

where ∗ indicates the convolution operation and V are the convolution layer weights.

### Backpropagation in dense connections: weight propagation

Consider the loss function to be H. The loss gradient in relation to an earlier layer's output y_i_ (i < m), where y_i_ must take into account any later layers that employ:

8 $$\frac{\delta H}{\delta yi}=\sum\nolimits_{k=i+1}^{M}\frac{\delta H}{\delta yi}. \frac{\delta yk}{\delta yi}$$

By doing so, training stability is increased and disappearing gradients are decreased by allowing continuous flow of gradients to layers prior to them.

### Utilizing adam optimizer for optimization

For our trials, we first set the Adam optimizer's usual decay of weights (L2 regularization) to 0.0001, the batch size to 32, and the learning rate to 0.001. These values were chosen using previous research and practical best practices. We used a grid search strategy across a constrained, small parameter space to fine-tune these hyperparameters. In particular, the batch size was assessed at values (64, 32, 16), and the learning rate was adjusted between (0.01, 0.001, 0.0001). To guarantee convergence and generalization, each configuration was evaluated using validation loss and accuracy trends. We made sure that the chosen combination produced steady training and optimal performance; however, we did not use genetic algorithms or Bayesian optimization due to computational resource limitations. The Adam optimizer, which uses estimations of the initial and subsequent moments of gradients to update parameters, is used to optimize the DenseNet-16 model.

- Θ_t_ = Model parameters.
- h_t_ = δ _Θt_M(Θ_t_) gredient.
- v = variance.
- m = mean.

9 $$\text{v}t\hspace{0.17em}=\hspace{0.17em}{\upalpha}_{2}.{\text{v}}_{\text{t}}-1\hspace{0.17em}+\hspace{0.17em}(1- {\upalpha}_{2}).{h}_{t}^{2}$$

10 $${\text{m}}_{\text{t}}\hspace{0.17em}=\hspace{0.17em}{\upalpha}_{1}.{\text{m}}_{\text{t}-1}\hspace{0.17em}+\hspace{0.17em}(1- {\upalpha}_{1}).{\text{h}}_{\text{t}}$$

11 $${v}_{t}^{\prime}=\frac{\text{vt}}{1- {\upalpha}_{2}^{t}}$$

12 $${m}_{t}^{\prime}=\frac{\text{mt}}{1- {\upalpha}_{1}^{t}}$$

13 $${\Theta }_{\text{t}+1}={\Theta }_{\text{t}}-\upalpha .\frac{{m}_{t}^{\prime}}{\sqrt{{v}_{t}^{\prime}}+ \varepsilon }$$

Here, A little constant ε keeps division by zero from happening, the decay rates for moment estimations are denoted by $\upalpha$_1_ and $\upalpha$_2_, which are typically 0.9 and 0.999. The nucleotide sequences (which are made up of the letters A, T, C, and G) need to be first quantitatively coded in order to convert the entire sequence into a logical representation. This is usually done by learning embeddings or k-mer embedding and the one-hot encoding method. This is necessary in order to customize DenseNet for genome-wide data. In order to allow DenseNet's two-dimensional layer of convolution to identify motifs (specific patterns) within the sequence, these decoded patterns are subsequently transformed into a two-dimensional matrix that resembles a picture. A 1,000-base sequence, for instance, could be rearranged to form an array like 225 × 225 × 3, where 3 stands for the number of nucleotide channels. Then, by altering the layers that provide input and output in accordance with the biological function—such as regulatory area identification, variation classification, or mutation effect prediction—DenseNet could be used directly from through or scratch transfer learning.

ImageNet pretrained weights were used to initialize the DenseNet-16 model. We preserved mid-level and low-level data on images by freezing the first three dense blocks and the primary layers in order to maximize the transfer learning process. Our chest X-ray dataset was used to unfreeze and fine-tune the last classifier and dense block layers. The technique of particular fine-tuning decreased overfitting and training time while enabling the trained model to adjust to COVID-19-specific imagery sequences. For final classification, a new dense and flattened layer (using softmax and dropout) and a median pooling layer were added.

## Result and discussion

The study used two datasets, the first of which had 2,100 chest X-ray pictures to serve as training purpose and 910 as testing purpose. Employing the NearKbest interpolation method, the photos were resized to a standard size of 225*225*3. DenseNet-16, a model framework with 20,311,999 parameters, was selected. We required 40 epochs were required for the model's training under the Anaconda Python environment. We use Batch Normalization following few layers to improve the performance of deep learning system. We used another NSBI dataset from Genbank for the purpose of this study, which contains genomic data for COVID 19 in FASTA format from different nations. We used the COVID 19 genome dataset from two separate countries. This keeps everything steady as you practice. The Adam optimizer, that is effective at determining the optimal parameters for simulation, that instructed to be used while configuring the model. This model learns more quickly and performs better because to this combination.

We calculated the genome sequences'overall computation time in 1.83 s and calculated the proportion of altered genome sequences at 1.31% (see Tables 2 and 3). Out of all genome sequences, COVID-19 was accurately categorized by computation time. The changing percentage represents the percentage of modified genomic sequences during COVID-19.

**Table 2:** Calculate computation time of genome sequence

Genome Sequence Analysis	
Model	Computation Time in Sec
GenoDense-Net	1.83
SPM Approach	2.13

**Table 3:** Percentage of genome sequence

Changing Percentage of Genome Sequence	
Model	Percentage
GenoDense-Net	1.31
SPM Approach	-

**Fig. 3:**
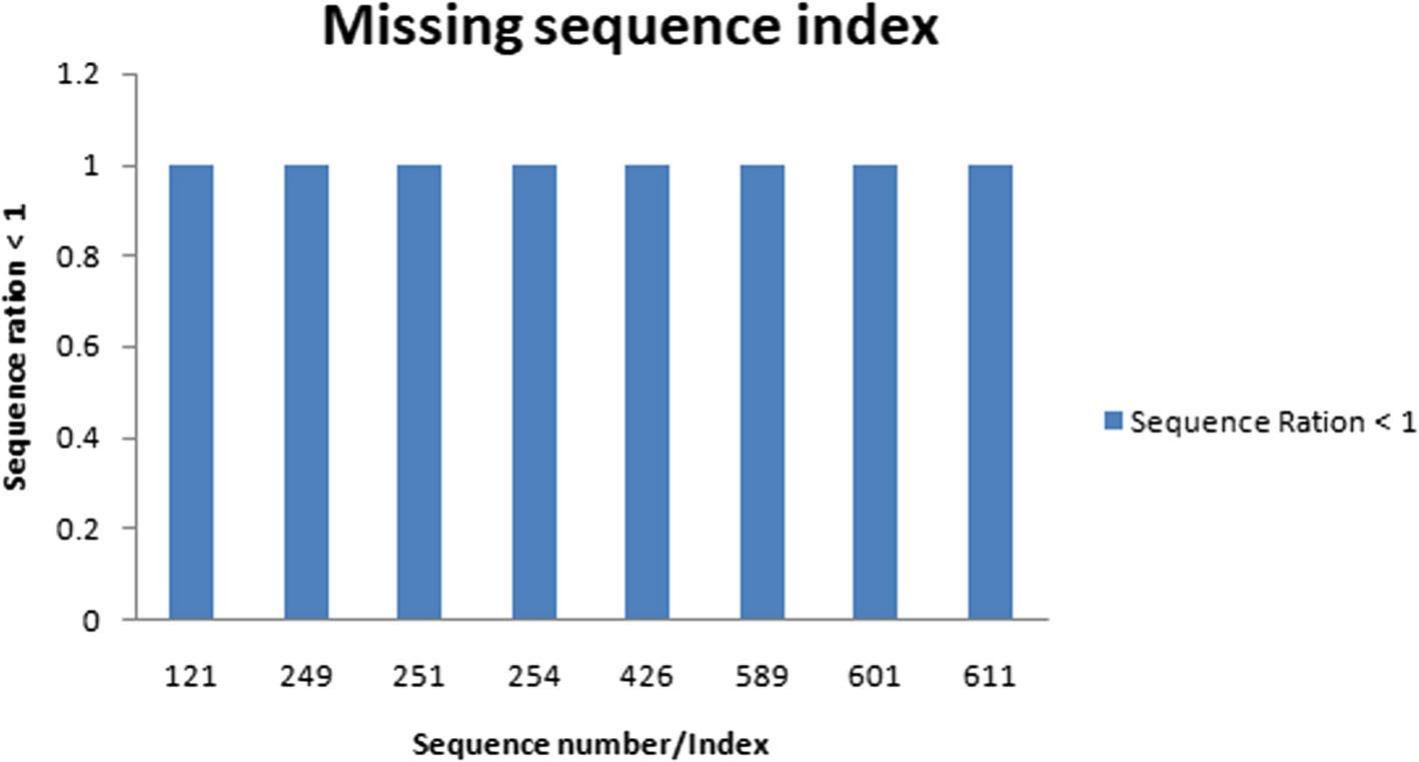
Missing index of genome sequence

The information from the DataFrame sequence''sequence ratios'column will be used to generate a bar graph. Sequence Ratio is written on the plot's y-axis as its label and"Index/Sequence numbering where genomes are missing"is representing by the x-axis label of the plot as shown in Fig. 3. The bar design can pinpoint certain indices or sequence numbers where genomes are absent. There may be clusters, voids, or patterns in the missing data that may be seen by visually examining the plot. This knowledge might be very helpful in comprehending the causes of missing genomes and perhaps direct additional research. The distribution pattern of sequencing proportions smaller than one may be shown and examined using the code, which is helpful overall. In addition, it facilitates data comparison, pattern identification, and communication. These details might be helpful in various sectors, including genetics, data analysis, research, and bioinformatics, wherever it's crucial to comprehend the sequence ratio's distribution.

The nucleotide percentages in the genome sequence (A, G, T, and C) are calculated using the code. The genome's makeup may be understood using this information, which is also useful for understanding the structural and functional characteristics of the genome. This programme also determines the GC content, representing 37.86% of the genome sequence's Guanine (G) followed by Cytosine (C) bases. GC concentration is a crucial metric in genomics because it can affect evolutionary trends, protein-coding competence, and DNA steadiness. That is utilized in a gene forecast, variety of genomic research, phylogenetic learning, and primer design.

The provided algorithm compares sequences of genes from the USA versus China to analyze them. A set of the names of genes is iterated over and over as several procedures are carried out for each gene. The genetic sequence is translated from DNA into RNA, transformed into a numpy array, and then plotted. The next step involves:

• Comparing the gene sequences from the USA and then China.
• Finding mutations.
• Storing the information about those mutations in a lexicon.

The function outputs the finished protein sequence once the mRNA sequence is translated. Overall, it makes gene mutation analysis easier and sheds light on the genetic variances from the USA to China.

**Fig. 4:**
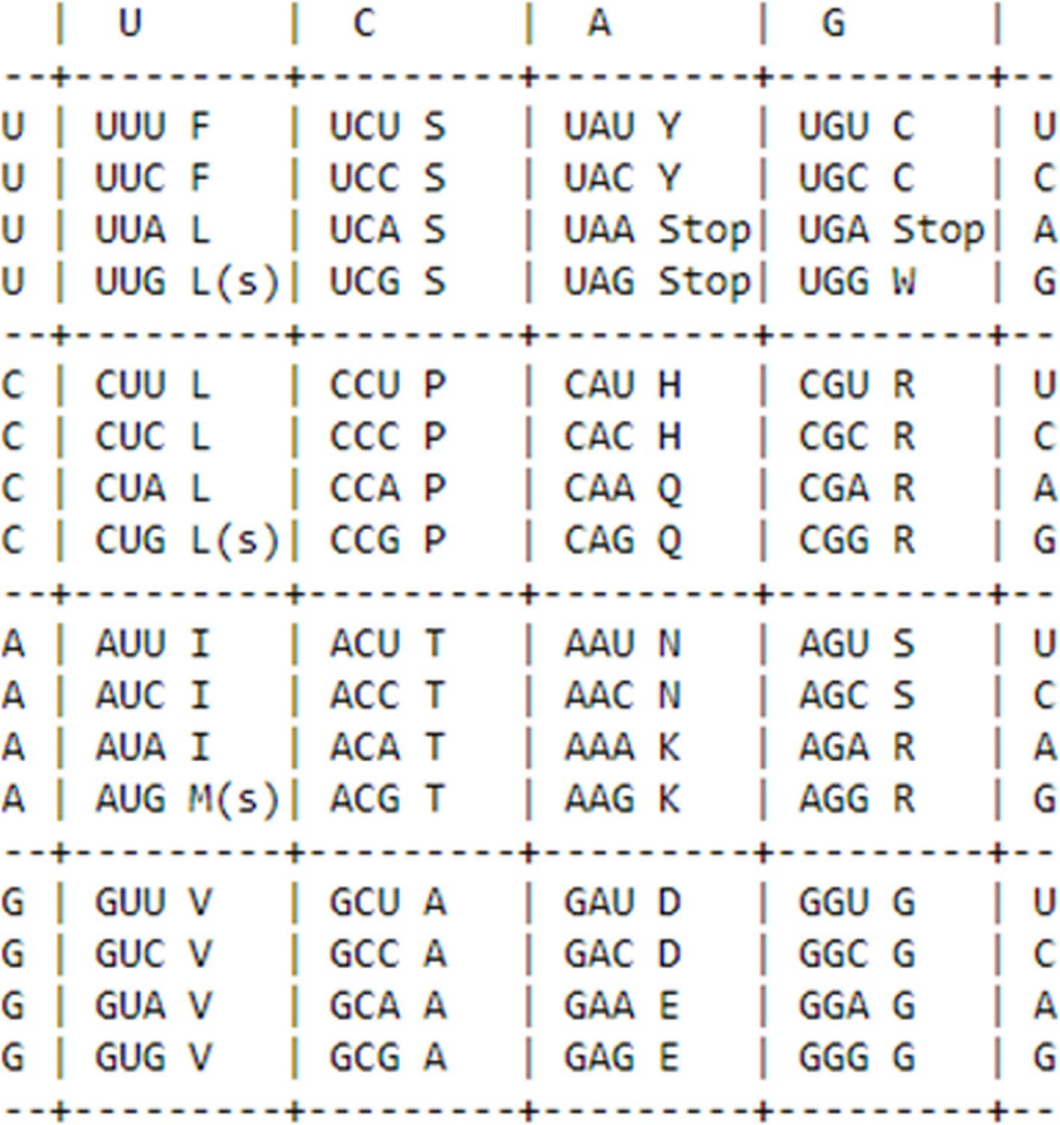
Mutation of genome sequence

The proposed technique emphasizes the translation of protein and mRNA analyses. It uses a specific translation table to translate an mRNA to an amino acid sequence. The length and the outcome of the amino acid sequence are printed. The model also prints the original mRNA sequence's length. Finally, it offers details on the typical RNA codon table. This code may be used to investigate protein production, comprehend how the genetic code is translated and examine sequences of proteins as shown in Fig. 4.

The number of distinct amino acids found in the sequence of proteins, some of which are present on more occasions than others, has been shown by counting everyone. The proportion of every amino acid in the protein's sequence provides a normalized representation, illuminating the similarities and differences between amino acids. Understanding the general amino acid distribution of the protein and spotting any biases or trends may be done using this information.

The protein's molecular weight indicates its dimensions and level of complexity. When comparing proteins or forecasting their physical characteristics, it indicates the total separate weight of every amino acid in the sequence. The aromaticity value (see Table 4) indicates the amount of aromatic amino acids in the protein sequence, including tryptophan, tyrosine, and phenylalanine. The presence of certain aromatic amino acids may have an impact on the protein's interactions, function, or structure if the aromaticity score is higher.

**Table 4:** Aromaticity and molecular mass of genome sequence

Variable	Value	
	GenoDense-Net	SPM Approach
Aromaticity (the degree to which molecules are aromatic)	0.107384441939120639	-
Molecular mass (weight within base pair)	794,048.92579999659	-

**Fig. 5:**
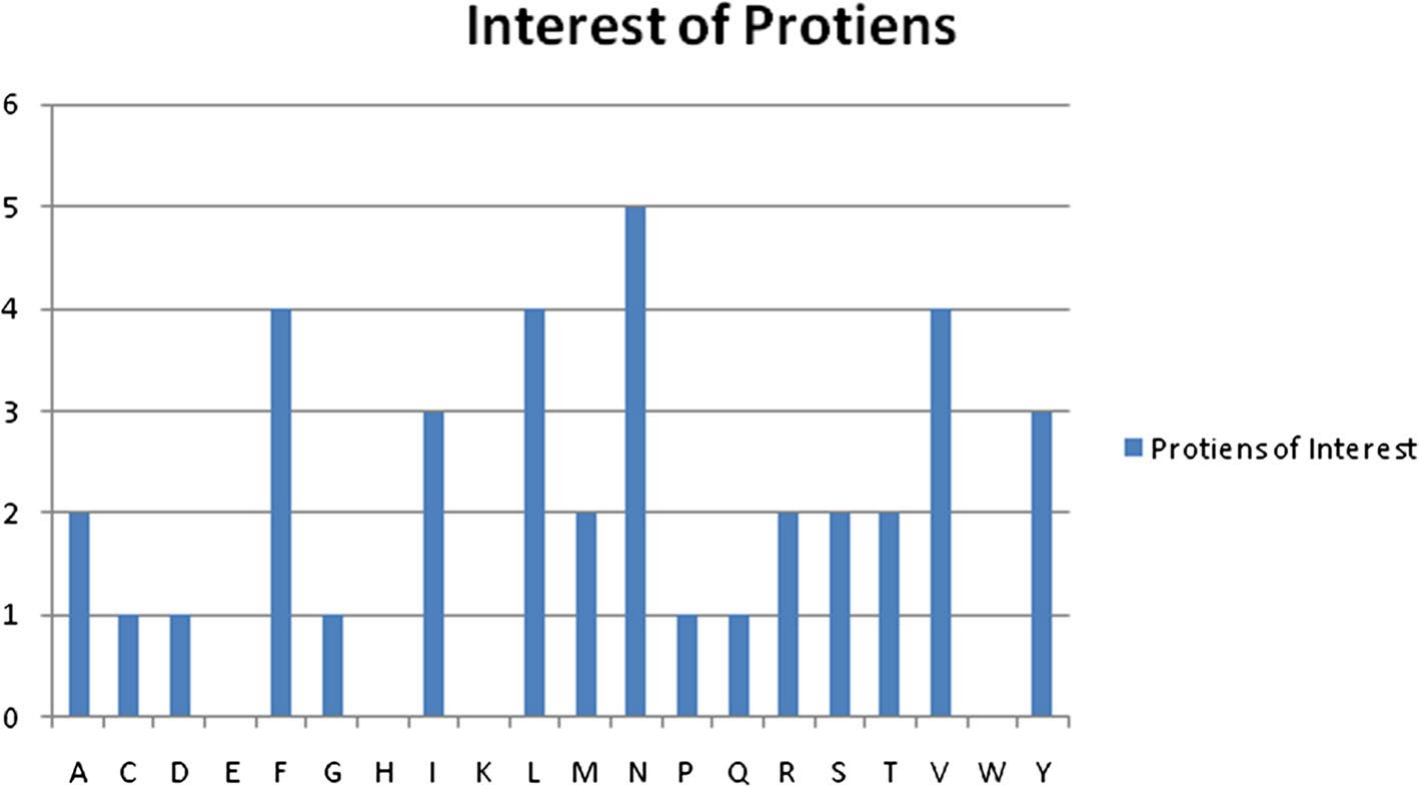
Interest of proteins of genome sequence

The GenoDense model creates a visualization showing the distribution or composition of a certain information collection in Fig. 5. In this instance, the proposed model precisely depicts the protein amino acid structure of the POI (Protein of Interest). The corresponding presence or quantity of each amino acid in the POI has been intuitively and easily visualized using a graph to illustrating the data. These may be helpful in different biochemical and biological investigations and can aid in understanding the qualities and traits of the protein.

**Fig. 6:**
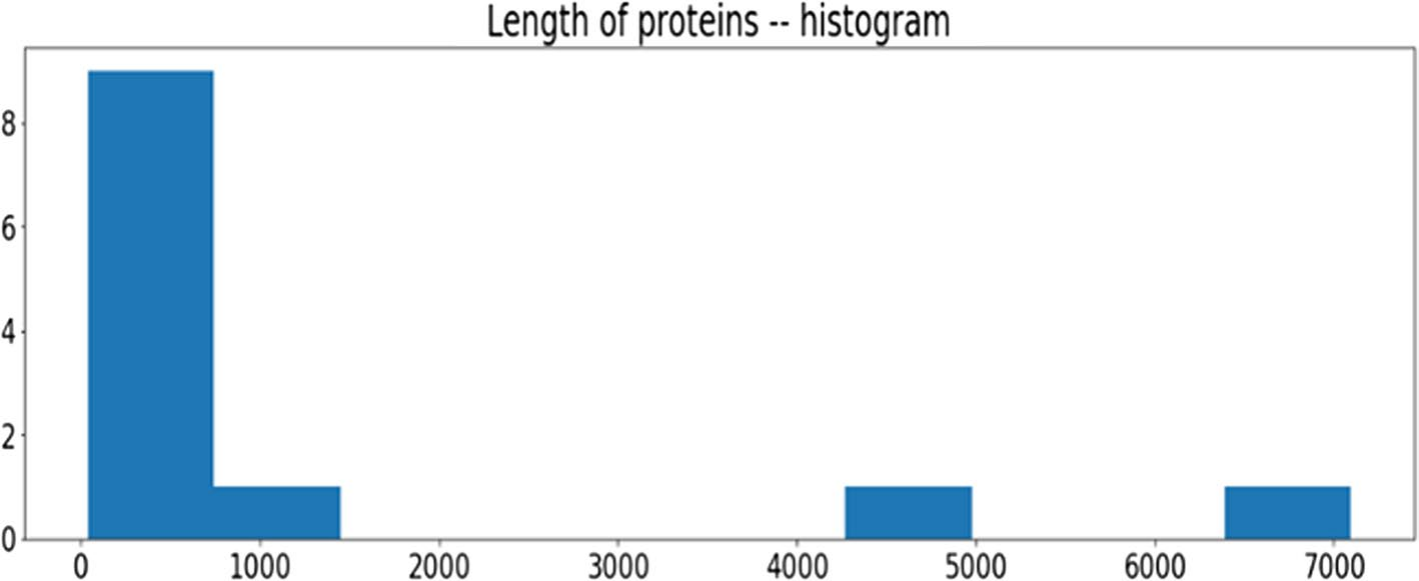
Length of proteins of genome sequence

The provided data also creates a graph which is showing the protein length variation. Our understanding of the distribution and frequency of various protein lengths in the data set is made possible through the proposed method's ability to visualize protein lengths. This Fig. 6 is a graph that visually depicts the protein length's distribution by displaying the protein's number concentrated and a range of lengths in each length bin. This information might be helpful for analysis and evaluation of the acquired understanding of the protein data under review and the spot patterns.

**Fig. 7:**
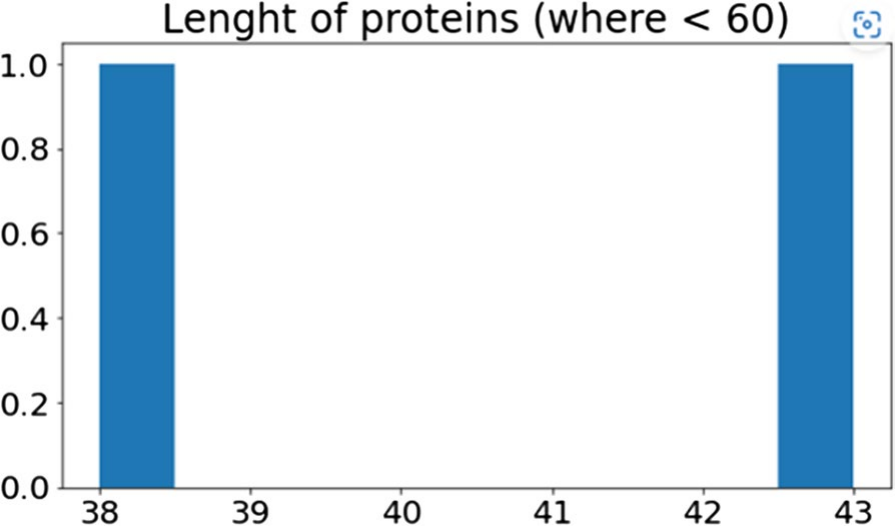
Length of proteins of genome sequence where threshold value < 60

To visualize, the residual length of range using a Fig. 7, the accompanying figure excludes the highest and lowest values or outliers from the protein's length data. The"functional_proteins"dataset is filtered by the algorithm, which only chooses proteins with lengths under 60. A new data frame contains these filtered proteins.

The protein length's distribution less than 60 kDa has displayed by plotting the graph employing the filtering protein's lengths. The visualization aids in understanding the protein's frequency or concentration together with shortened lengths and the overall distribution of protein lengths within a certain range. Insights into the lengths of common or normal proteins of the dataset may be gained by performing a targeted analysis of the protein length distribution free from the impact of extreme values.

**Fig. 8:**
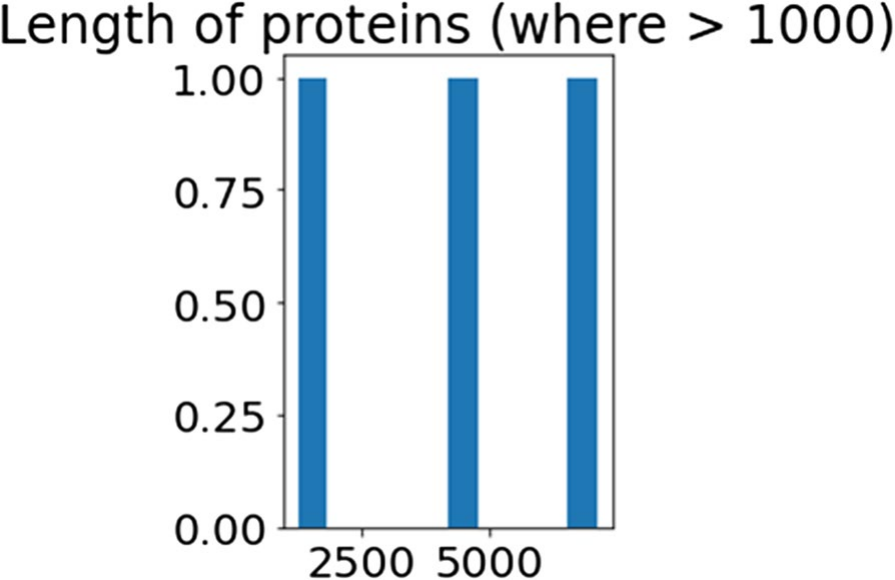
Length of proteins where threshold value > 1000

The"functional_proteins"dataset is filtered using the provided code to only include proteins with a length larger than 1000 kDa. A new data frame contains the proteins that have been filtered. The method then creates a histogram displaying the distribution of proteins with lengths larger than 1000, employing the processed proteins'total lengths. The distribution of greater lengths of protein throughout the data set is shown visually by the Fig. 8.

By examining the visualization, one can gain understanding about the concentration and frequency of proteins of longer chains, which can help one comprehend the structure of the huge proteins inside the data set. The visualization aids in the discovery of any patterns or traits connected to proteins with lengths greater than the predetermined threshold of 1000.

Pairwise sequencing of the connections between various DNA sequences is done using the suggested technique. It determines the alignment scores between the sequences. It divides each by the total length of the sequence used as a reference to get similarities scoring between the sequences and turns those into percentages.

**Fig. 9:**
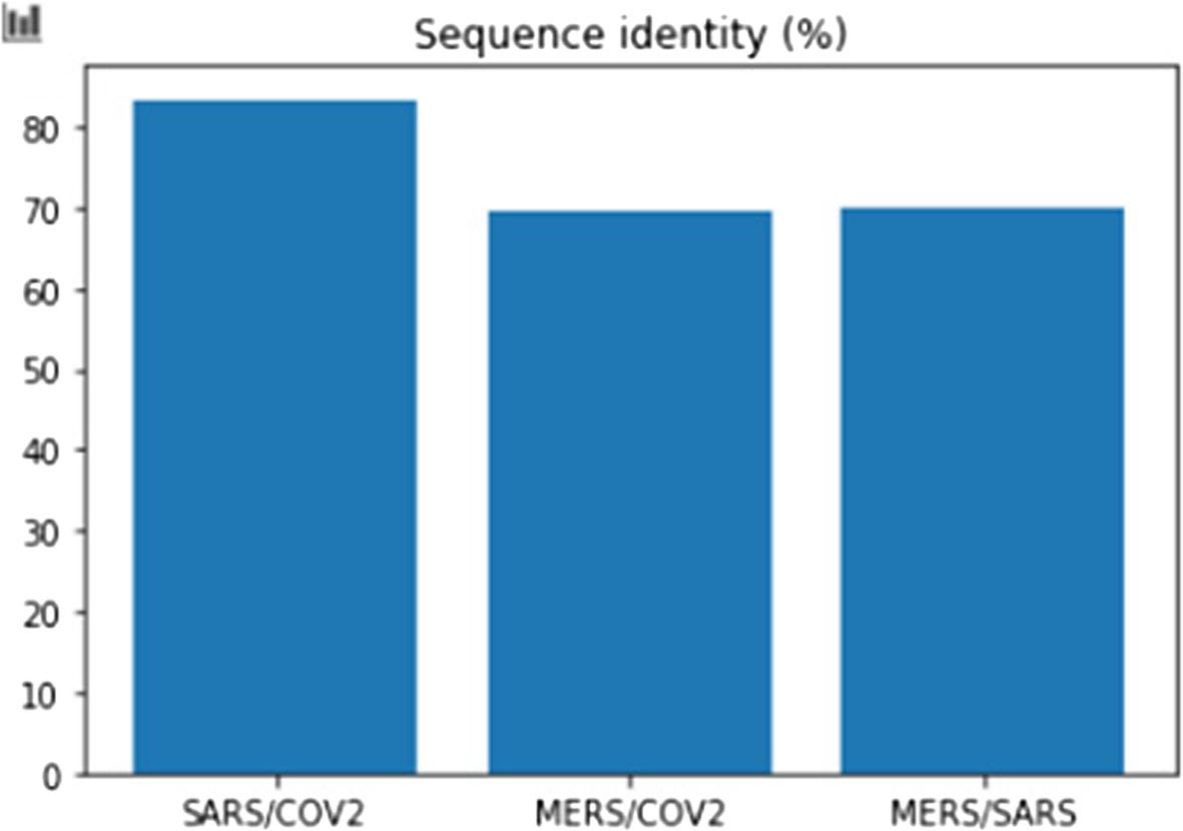
Sequence identity in percentage of genome sequence

Then, three comparisons— MERS/SARS, MERS/COV2, and SARS/COV2 —has been presented with the computed similarity percentages. The pattern of similarities between various viruses, has presented in the Fig. 9, is shed light on by these comparisons. In contrast to the Y-axis which shows the proportion of sequence identity, the X-axis displays various sequence comparisons. Figure 9 makes it easy to compare the sequence resemblances of the various DNA sequences. To analyse DNA sequences, the technique combines pairwise sequence position, visualizing DNA sequences, and computing relevance scores.

**Fig. 10:**
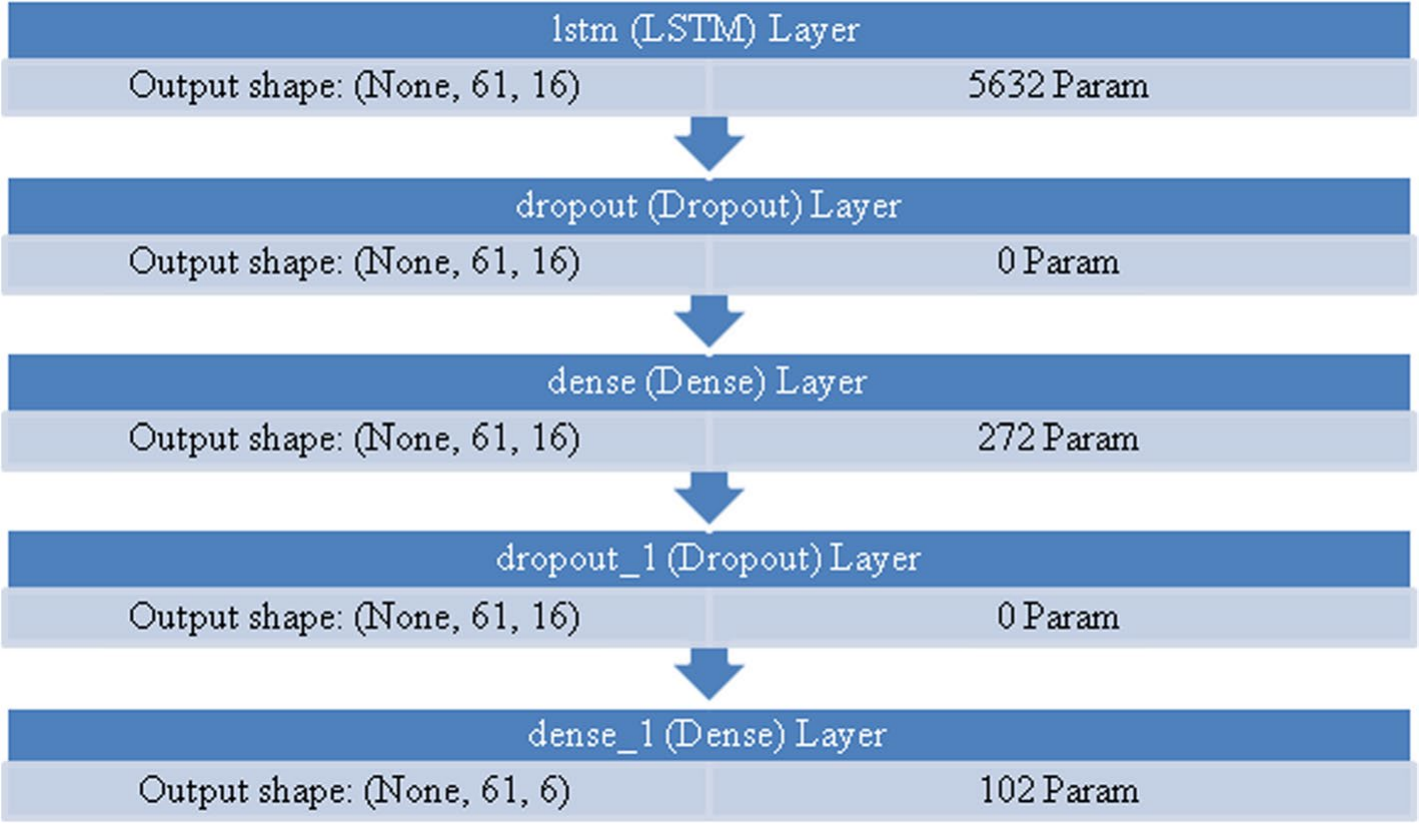
Multi-layered neural network structures

A multi-layered neural network structure is depicted in the Fig. 10. The Dense layer, a Dropout layer, and the LSTM layer are all parts of the model architecture. According to the LSTM layer's output structure (None, 61, 16), it accepts sequences of inputs of length 61 and generates sequences of output of length 16. There are 5632 parameters in the LSTM layer.

The output shape is unaffected by the Dropout layer, which operates for regularisation and to avoid overfitting. Since it doesn't add additional weights to the structure, it has no parameters. The output shape of the Dense layer, which comes after the Dropout layer, is (None, 61, 16). The supplied data is transformed using the 272 parameters that are introduced. A second Dropout layer is added without altering the output form. The model is finished using a Dense layer with the output form (None, 61, 6). The 102 parameters have been used for this layer. The model comprises 6,006 distinct parameters in total, every one of that is trainable. The parameters that can be trained are modified to enhance the performance of the model (see Table 5). These non-trainable parameters of model are set to 0. The design of the model, the variable’s count for every layer, along with the form of the result at each level are all described in this overview. It aids in comprehending the neural network model's intricacy and capabilities. Adam, the optimizer, is configured by the code with particular weight decay and learning rate. During training, the optimize is in charge of adjusting the neural network's weights. Categorical cross-entropy, a popular loss function for multi-class classification issues, was selected as the loss function.

**Table 5:** The CNN model’s performance comparison

Model	Accuracy	Total Parameter (million)	Trainable Parameter (million)	Traning Time
ReseNet and AlexNet	49	61.23	2.46	51
VGG19	92	143.21	20.01	59
DenseNet169	96.37	25.13	23.40	65
VGG16	50.55	138.37	14.69	55
GenoDense-Net	99.18	20.32	7.66	63

Every training development of each epoch in the training and validation of model is valuable. The information is broken out as follows; Epoch: When training for this particular epoch, the loss function's value is called the loss. It displays the model's performance and shows how closely the predictions match the actual numbers, and Accuracy: The model's accuracy for the training data set represents up to this present epoch. It displays the percentage of accurately predicted samples. Several of the test models (VGG-19, ResNet-50, AlexNet, and VGG-16) were developed applying transfer learning techniques with previously trained ImageNet parameters in order to guarantee an even evaluation. Binary classification was used for the last fully connected layers. The training setup (batch size = 32, learning rate = 0.001), number of epochs (40), and data preparation were identical for every model. The Google Colaboratory with GPU support (identical hardware environment) was used for the training. A comparison of training efficiency, model complexity, and performance is shown in Table 5. In particular, we used pairwise t-tests (with Bonferroni correction) after a one-way ANOVA test to assess the significance of the performance differences between the previous models and GenoDense-Net. Accuracy scores gathered from five separate runs of each model utilizing identical train-test splits have been used for the tests. The ANOVA findings verified that the mean accuracy of the models varied statistically significantly (p < 0.01). Furthermore, GenoDense-Net's improvements over all other models are statistically significant, according to the t-tests (p < 0.05 after correction) (see Table 6).

**Table 6:** The CNN model’s statistical comparison

Model	*p*-value vs t-test	Standard Deviation	Statistical Significant
ReseNet and AlexNet	< 0.001	1.13	Significant
VGG19	0.002	0.96	Significant
DenseNet169	0.003	1.05	Significant
VGG16	< 0.001	1.10	Significant
GenoDense-Net	-	0.33	-

Additionally, we have emphasized the special contribution of GenoDense-Net, a hybrid model that combines an image-inspired DenseNet structure with genome-wide coding to enable high accuracy (99.18%) with the classification of infectious diseases from DNA sequences while preserving computational clarity. This comparison makes our work's positioning stronger (see Table 7) and makes clear how original it is compared to probabilistic and transformer-based sequence models. The model’s complexity comparison is presented in Table 8.

**Table 7:** GenoDense-Net compared to the most advanced genomic models of AI

Model	Task	Architecture	Performance
GenomeBERT	Annotation of the genome and prediction of variation effects	BERT (Transformer)	F1-score ≈ 93%–97%
DeepSEA	Chromatin characteristics and the prediction of regulatory regions	CNN	AUC ≈ 95%
DeepVariant	Calling SNPs and indel variants	Multi-task learning on CNN	Precision ≈ 99.49%
DanQ	Prediction of functional regions	BiLSTM + CNN	AUC ≈ 94%
GenoDense-Net	Classification of infectious diseases (e.g., COVID-19)	DenseNet-16 (Transfer Learning + CNN)	Accuracy = 99.18% F1-score: 99.11% Precision: 98.96% Recall: 98.32% MCC: 0.979 AUC-ROC: 0.989

**Table 8:** The CNN Model’s complexity comparison

Model	Inference time per sample in ms	Training time per epoch in s	Memory usage in GB
ReseNet and AlexNet	5.3	36	2.6
VGG19	5.5	66	10.2
DenseNet169	4.7	57	8.2
VGG16	9.6	64	5.2
GenoDense-Net	3.2	43	6.7

**Fig. 11:**
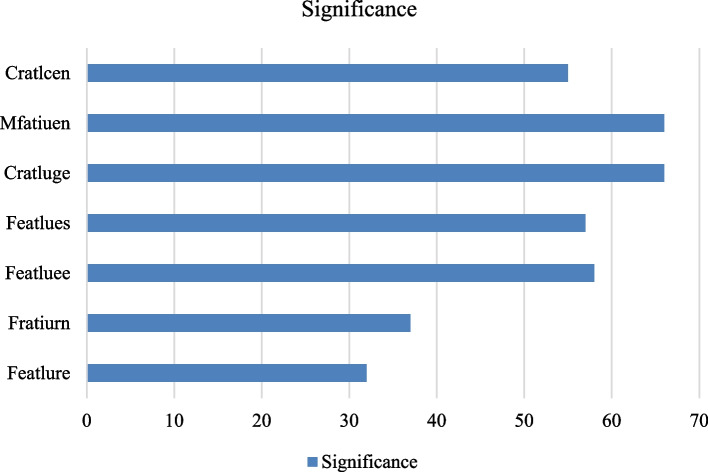
Feature’s significance

A standard bar plot is depicted in Fig. 11, where each bar's length indicates how important a particular feature is. The'Features'axis in this figure would stand for image features (such as certain patterns or areas in the X-ray pictures), and the'significance'axis represents the relative contribution of each feature to the prediction of the model.

**Fig. 12:**
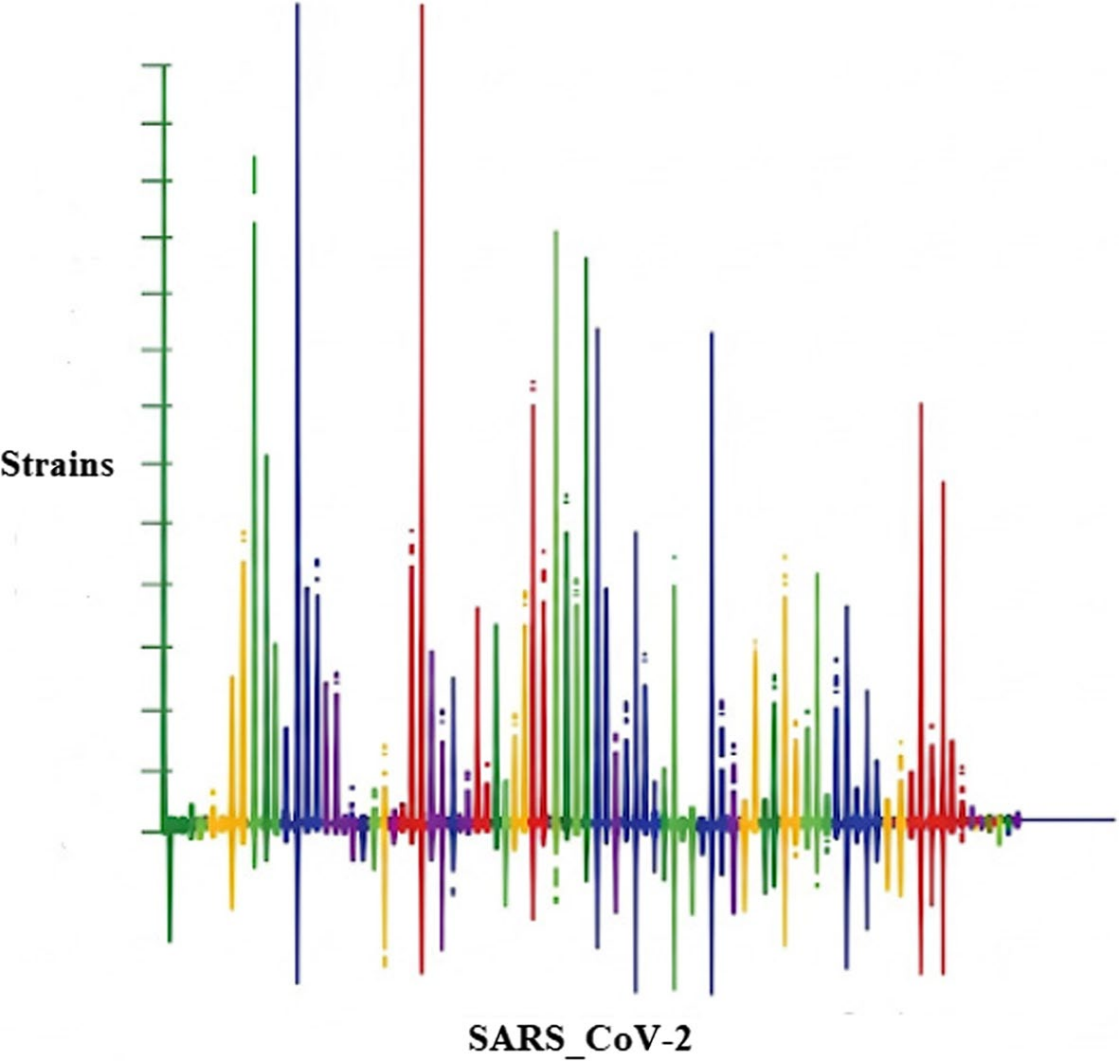
Genetic variations among strains

Figure 12 shows the genetic differences between different strains of COVID-19. These many strains are listed on a vertical axis in the picture, while particular genes or areas of the viral genome are indicated on a horizontal axis. Each strain is given a unique hue in the depiction, making it simple to compare their genetic variations and giving a clear picture of the strains'genomic diversity.

## Conclusion

In this study, we devised a method for determining a patient's level of infectiousness from their genome sequence using the DenseNet-16 model. We employed a transfer learning strategy after using a pre-trained DenseNet-16 network. Data preprocessing was done using the NearKbest interpolation approach, and optimization was done using Adam Optimizer. Our accuracy was 99.18%. Additionally, we discussed incomplete proteins, sequence identity as a proportion of the genome's sequence, protein length, aromaticity and molecular mass, mutations in the genome's sequence, and a missing index of the genome's sequence. We outperformed ResNet-50, AlexNet, VGG-19 models, and VGG-16 in terms of accuracy. Future studies should work to close this gap in order to guarantee the model's efficacy and moral application in medical facilities. We have focus on performing an additional thorough validation of clinical research with a bigger, more varied dataset sourced from various medical facilities. We can work on assessing the effectiveness of the model over a range of segments of the population, genders, comprising multiple age brackets, and racial/ethnic groupings, to guarantee that it can be generalized. Also evaluating how the framework affects medical choices, outcomes for patients, and the use of medical resources. Also, we can work on resolving transparency and moral issues in the application of artificial intelligence in medical. Although the suggested methodology is highly accurate at identifying COVID-19 using chest X-ray pictures, there are difficulties when implementing it in actual clinical situations. Careful consideration must be given to variations in patient demographics, imaging conditions, and interaction with medical facilities. To guarantee responsible AI use, ethical issues including model bias, data privacy,and decision openness also need to be addressed. Crucially, our findings are based on a small dataset, and before using the system in standard medical practice, external validation utilizing a variety of multi-center medical data is essential.

## Data Availability

No datasets were generated or analysed during the current study.
